# 1-(*o*-Tol­yl)thio­urea

**DOI:** 10.1107/S1600536808024161

**Published:** 2008-08-06

**Authors:** Rodrigo S. Corrêa, Leandro Ribeiro, Javier Ellena, Osvaldo Estévez-Hernández, Julio Duque

**Affiliations:** a Grupo de Cristalografía, Instituto de Física de São Carlos, Universidade de São Paulo, São Carlos, Brazil; b Department of Structure Analysis, Institute of Materials, Zapata & G, University of Havana, Cuba

## Abstract

In the title compound, C_8_H_10_N_2_S, the *o*-tolyl group and the thio­urea core are planar. The mean planes of the two groups are almost perpendicular [82.19 (8)°]. The thio­urea group is in the thio­amide form, in which resonance is present. In the crystal structure, mol­ecules are linked by inter­molecular N—H⋯S hydrogen bonds, forming two infinite chains parallel to the (110) and (1
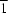
0) planes.

## Related literature

For general background, see: Koketsu & Ishihara (2006 ▶); Struga *et al.* (2007 ▶). For related structures, see: Corrêa *et al.* (2006 ▶); Corrêa *et al.* (2008 ▶); Estévez-Hernández *et al.* (2008 ▶); Duque *et al.* (2008 ▶). For the synthesis, see: Otazo-Sánches *et al.* (2001 ▶). For related literature, see: Otazo *et al.* (2001 ▶); Ramadas *et al.* (1998 ▶).
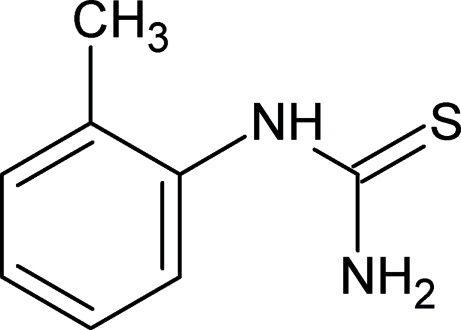


## Experimental

### 

#### Crystal data

C_8_H_10_N_2_S
*M* *_r_* = 166.25
Monoclinic, 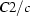

*a* = 15.1323 (3) Å
*b* = 7.7965 (2) Å
*c* = 15.3222 (4) Å
β = 90.828 (2)°
*V* = 1807.61 (8) Å^3^
*Z* = 8
Mo *K*α radiation
μ = 0.30 mm^−1^
*T* = 294 K
0.31 × 0.22 × 0.10 mm

#### Data collection

Nonius KappaCCD diffractometer
Absorption correction: gaussian (Coppens *et al.*, 1965 ▶) *T*_min_ = 0.973, *T*_max_ = 0.991
6748 measured reflections
1914 independent reflections
1438 reflections with *I* > 2σ(*I*)
*R*_int_ = 0.031

#### Refinement

*R*[*F* ^2^ > 2σ(*F* ^2^)] = 0.046
*wR*(*F* ^2^) = 0.140
*S* = 1.03
1914 reflections
101 parameters
H-atom parameters constrained
Δρ_max_ = 0.19 e Å^−3^
Δρ_min_ = −0.21 e Å^−3^

### 

Data collection: *COLLECT* (Nonius, 2000 ▶); cell refinement: *SCALEPACK* (Otwinowski & Minor, 1997 ▶); data reduction: *DENZO* (Otwinowski & Minor, 1997 ▶) and *SCALEPACK*; program(s) used to solve structure: *SHELXS97* (Sheldrick, 2008 ▶); program(s) used to refine structure: *SHELXL97* (Sheldrick, 2008 ▶); molecular graphics: *ORTEP-3 for Windows* (Farrugia, 1997 ▶) and *Mercury* (Macrae *et al.*, 2006 ▶); software used to prepare material for publication: *WinGX* (Farrugia, 1999 ▶).

## Supplementary Material

Crystal structure: contains datablocks global, I. DOI: 10.1107/S1600536808024161/fb2105sup1.cif

Structure factors: contains datablocks I. DOI: 10.1107/S1600536808024161/fb2105Isup2.hkl

Additional supplementary materials:  crystallographic information; 3D view; checkCIF report

## Figures and Tables

**Table:** 

C1—N2	1.321 (2)
C1—N1	1.329 (2)
C1—S1	1.6868 (18)
C2—N1	1.435 (2)

**Table:** 

N2—C1—N1	117.34 (16)
N2—C1—S1	121.63 (14)
N1—C1—S1	121.03 (13)

**Table 2:** Hydrogen-bond geometry (Å, °)

*D*—H⋯*A*	*D*—H	H⋯*A*	*D*⋯*A*	*D*—H⋯*A*
N1—H1⋯S1^i^	0.86	2.53	3.368 (2)	165
N2—H2*B*⋯S1^ii^	0.86	2.52	3.362 (2)	166

Symmetry codes: (i) 
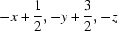
; (ii) 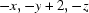.

**Fig. 1.**
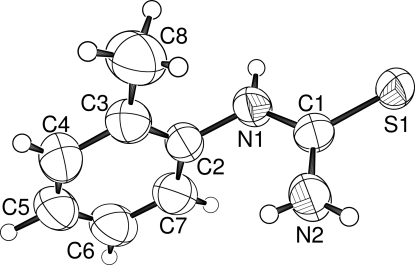
The title molecule with the atom-numbering scheme. The displacement ellipsoids are drawn at the 50% probability level and the H atoms are shown as small spheres of arbitrary radii.

**Fig. 2.**
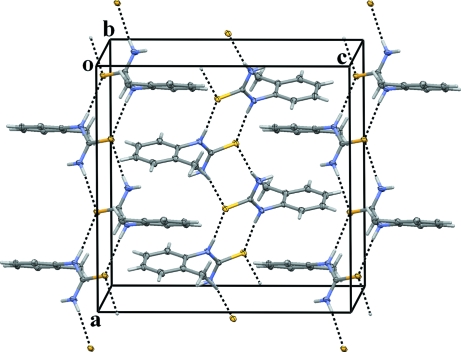
Representation of the chains linked by N—H···S hydrogen bonds.

**Fig. 3.**
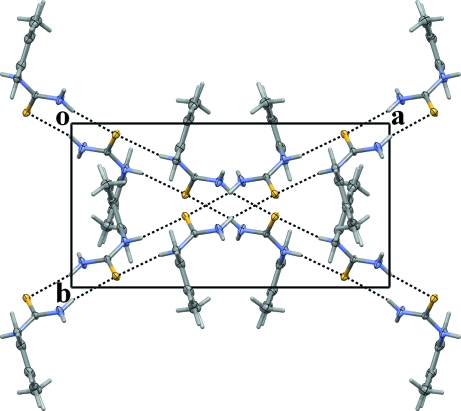
Crystal packing view along c axis showing the two independent chains.

## supplementary crystallographic information

### Comment

Thiourea itself as well as its derivatives are known to be biologically active (Koketsu & Ishihara, 2006). Their antimicrobial, cytotoxic and anti-HIV activities have been recently tested (Struga *et al.*, 2007). Also, *ortho*-substituted aromatic thiourea derivatives have received special attention because of their fungicidal activity (Ramadas *et al.*, 1998). The reaction of the furoyl isothiocyanate with *o*-toluidine in dry acetone yielded the title compound, *N*-(*o*-tolyl) thiourea, as a secondary product (Fig. 1).

The title molecule is present in the thioamide form and it is a typical N-monosubstituted thiourea derivative with usual geometric parameters. The C—S bond [1.687 (2) Å] shows the expected double-bond character. The short bond-lengths of the C1—N1 [1.329 (2) Å] and C1—N2 [1.321 (2) Å] indicate partial double bond character, similarly to other thiourea derivatives where electron delocalization in the N—C—S moiety is present (Corrêa *et al.*, 2008; Estévez-Hernández *et al.*, 2008, Duque *et al.*, 2008). In addition, the values of the bond angles that are close to 120° also suggest the resonance effect.

As might be expected both the central thiourea fragment as well as the *o*-tolyl group are planar. The largest deviation from the least square plane through the seven atoms of the *o*-tolyl group occurs for the atom C2 [displacement = 0.0038 (15) Å], with a r.m.s. deviation of 0.0022 Å for all the carbons in the *o*-tolyl group. In the thiourea fragment, the largest displacement is for the atom C1 [0.001 (1) Å], with a r.m.s. deviation of 0.003 Å. The *o*-tolyl group is almost perpendicular to the plane formed by the thiourea molecule (82.19 (8)°).

Fig. 2 shows the arrangement of the molecules in the unit cell. In the crystal structure, the molecules are linked by N—H···S hydrogen bonds that stabilize the packing (Table 1). In the previous studies (Corrêa *et al.*, 2006; Corrêa *et al.*, 2008) have been reported the N—H···S interactions with the formation of the centrosymmetric dimers. In contrast to these structures, in the present structure these intermolecular interactions form two independent chains parallel to the (110) and (1–10) planes (Figure 3).

### Experimental

The title compound was obtained as a secondary product during the synthesis of 1-(2-furoyl)-3-(*o*-tolyl) thiourea according to procedure described by Otazo-Sánchez *et al.* (2001) by converting furoyl chloride into furoyl isothiocyanate and then condensing with the appropriate 0-toluidine. The colourless prism-shaped single crystals were formed by slow evaporation from a methanol/acetonitrile (1:1) solution.

### Refinement

All the hydrogen atoms were located in the difference Fourier maps. Nevertheless,  they were situated in the idealized positions and refined using the riding-hydrogen model. N—H = 0.86 Å, C_aryl_—H = 0.93 Å, C_methyl_= 0.96 Å. *U*_iso_(H) = 1.2*U*_eq_(*N*,*C*_aryl_) or *U*_iso_(H) = 1.5*U*_eq_(C_methyl_).

### Crystal data

**Table:**

C_8_H_10_N_2_S	*F*_000_ = 704
*M**_r_* = 166.25	*D*_x_ = 1.222 Mg m^−^^3^
Monoclinic, *C*2/*c*	Mo *K*α radiation λ = 0.71073 Å
Hall symbol: -C 2yc	Cell parameters from 15611 reflections
*a* = 15.1323 (3) Å	θ = 2.9–26.7º
*b* = 7.7965 (2) Å	µ = 0.30 mm^−^^1^
*c* = 15.3222 (4) Å	*T* = 294 K
β = 90.828 (2)º	Prism, colourless
*V* = 1807.61 (8) Å^3^	0.31 × 0.22 × 0.10 mm
*Z* = 8	

### Data collection

**Table:**

Nonius KappaCCD diffractometer	1438 reflections with *I* > 2σ(*I*)
Radiation source: fine-focus sealed tube Enraf Nonius FR590	*R*_int_ = 0.031
Monochromator: horizontally mounted graphite crystal	θ_max_ = 26.8º
φ scans and ω scans winth κ offsets	θ_min_ = 3.8º
Absorption correction: gaussian(Coppens *et al.*, 1965)	*h* = −19→18
*T*_min_ = 0.973, *T*_max_ = 0.991	*k* = −9→9
6748 measured reflections	*l* = −19→19
1914 independent reflections	

### Refinement

**Table:**

Refinement on *F*^2^	Secondary atom site location: difference Fourier map
Least-squares matrix: full	Hydrogen site location: difference Fourier map
*R*[*F*^2^ > 2σ(*F*^2^)] = 0.046	H-atom parameters constrained
*wR*(*F*^2^) = 0.140	*w* = 1/[σ^2^(*F*_o_^2^) + (0.0896*P*)^2^ + 0.2205*P*] where *P* = (*F*_o_^2^ + 2*F*_c_^2^)/3
*S* = 1.03	(Δ/σ)_max_ < 0.001
1914 reflections	Δρ_max_ = 0.19 e Å^−^^3^
101 parameters	Δρ_min_ = −0.21 e Å^−^^3^
40 constraints	Extinction correction: none
Primary atom site location: structure-invariant direct methods	

### Special details

**Table:**

Geometry. All e.s.d.'s (except the e.s.d. in the dihedral angle between two l.s. planes) are estimated using the full covariance matrix. The cell e.s.d.'s are taken into account individually in the estimation of e.s.d.'s in distances, angles and torsion angles; correlations between e.s.d.'s in cell parameters are only used when they are defined by crystal symmetry. An approximate (isotropic) treatment of cell e.s.d.'s is used for estimating e.s.d.'s involving l.s. planes.

### Fractional atomic coordinates and isotropic or equivalent isotropic displacement parameters (Å^2^)

**Table:**

	*x*	*y*	*z*	*U*_iso_*/*U*_eq_	
C1	0.11314 (11)	0.8287 (2)	0.06714 (12)	0.0577 (5)	
C2	0.15409 (11)	0.6359 (3)	0.18736 (12)	0.0611 (5)	
C3	0.12742 (14)	0.4692 (3)	0.18788 (14)	0.0740 (6)	
C4	0.11722 (18)	0.3923 (3)	0.27051 (17)	0.0886 (7)	
H4	0.0995	0.2783	0.2737	0.106*	
C5	0.13277 (17)	0.4810 (4)	0.34545 (15)	0.0887 (7)	
H5	0.1256	0.4269	0.399	0.106*	
C6	0.15879 (16)	0.6486 (4)	0.34321 (15)	0.0884 (7)	
H6	0.1691	0.7089	0.3947	0.106*	
C7	0.16938 (15)	0.7262 (3)	0.26419 (14)	0.0769 (6)	
H7	0.187	0.8404	0.2618	0.092*	
C8	0.1108 (2)	0.3713 (4)	0.10584 (18)	0.1082 (9)	
H8A	0.0882	0.2598	0.1197	0.162*	
H8B	0.1651	0.3594	0.0748	0.162*	
H8C	0.0685	0.4317	0.0701	0.162*	
N1	0.16917 (10)	0.7203 (2)	0.10564 (10)	0.0665 (5)	
H1	0.218	0.6992	0.0797	0.08*	
N2	0.03568 (11)	0.8514 (3)	0.10408 (12)	0.0897 (7)	
H2A	0.0232	0.797	0.1512	0.108*	
H2B	−0.0023	0.9205	0.081	0.108*	
S1	0.14045 (3)	0.93276 (6)	−0.02514 (3)	0.0663 (2)	

### Atomic displacement parameters (Å^2^)

**Table:**

	*U*^11^	*U*^22^	*U*^33^	*U*^12^	*U*^13^	*U*^23^
C1	0.0496 (9)	0.0631 (10)	0.0607 (10)	0.0091 (8)	0.0069 (8)	0.0058 (8)
C2	0.0486 (9)	0.0738 (12)	0.0610 (11)	0.0136 (8)	0.0074 (8)	0.0143 (9)
C3	0.0718 (13)	0.0793 (14)	0.0711 (13)	0.0099 (11)	0.0082 (10)	0.0043 (10)
C4	0.0938 (16)	0.0817 (14)	0.0906 (17)	0.0062 (12)	0.0181 (14)	0.0225 (13)
C5	0.0963 (16)	0.1078 (18)	0.0624 (13)	0.0242 (15)	0.0148 (11)	0.0210 (13)
C6	0.0920 (16)	0.1113 (19)	0.0619 (13)	0.0199 (14)	0.0023 (11)	0.0010 (13)
C7	0.0741 (13)	0.0871 (14)	0.0696 (13)	0.0052 (11)	0.0053 (10)	0.0012 (11)
C8	0.130 (2)	0.1022 (18)	0.0930 (18)	−0.0140 (18)	0.0182 (17)	−0.0160 (16)
N1	0.0539 (8)	0.0834 (11)	0.0626 (9)	0.0202 (7)	0.0146 (7)	0.0205 (8)
N2	0.0616 (10)	0.1243 (16)	0.0838 (12)	0.0374 (10)	0.0250 (9)	0.0451 (12)
S1	0.0622 (4)	0.0697 (4)	0.0673 (4)	0.0189 (2)	0.0158 (2)	0.0183 (2)

### Geometric parameters (Å, °)

**Table:**

C1—N2	1.321 (2)	C5—H5	0.93
C1—N1	1.329 (2)	C6—C7	1.365 (3)
C1—S1	1.6868 (18)	C6—H6	0.93
C2—C3	1.361 (3)	C7—H7	0.93
C2—C7	1.388 (3)	C8—H8A	0.96
C2—N1	1.435 (2)	C8—H8B	0.96
C3—C4	1.411 (3)	C8—H8C	0.96
C3—C8	1.489 (3)	N1—H1	0.86
C4—C5	1.358 (4)	N2—H2A	0.86
C4—H4	0.93	N2—H2B	0.86
C5—C6	1.365 (4)		
			
N2—C1—N1	117.34 (16)	C5—C6—H6	120.5
N2—C1—S1	121.63 (14)	C6—C7—C2	120.5 (2)
N1—C1—S1	121.03 (13)	C6—C7—H7	119.8
C3—C2—C7	121.68 (19)	C2—C7—H7	119.8
C3—C2—N1	119.57 (19)	C3—C8—H8A	109.5
C7—C2—N1	118.73 (19)	C3—C8—H8B	109.5
C2—C3—C4	116.6 (2)	H8A—C8—H8B	109.5
C2—C3—C8	122.1 (2)	C3—C8—H8C	109.5
C4—C3—C8	121.4 (2)	H8A—C8—H8C	109.5
C5—C4—C3	121.5 (2)	H8B—C8—H8C	109.5
C5—C4—H4	119.3	C1—N1—C2	124.75 (15)
C3—C4—H4	119.3	C1—N1—H1	117.6
C4—C5—C6	120.9 (2)	C2—N1—H1	117.6
C4—C5—H5	119.6	C1—N2—H2A	120
C6—C5—H5	119.6	C1—N2—H2B	120
C7—C6—C5	119.0 (2)	H2A—N2—H2B	120
C7—C6—H6	120.5		
			
C7—C2—C3—C4	−0.8 (3)	C5—C6—C7—C2	−0.1 (3)
N1—C2—C3—C4	177.56 (18)	C3—C2—C7—C6	0.7 (3)
C7—C2—C3—C8	180.0 (2)	N1—C2—C7—C6	−177.68 (18)
N1—C2—C3—C8	−1.7 (3)	N2—C1—N1—C2	−4.6 (3)
C2—C3—C4—C5	0.4 (3)	S1—C1—N1—C2	175.28 (16)
C8—C3—C4—C5	179.6 (3)	C3—C2—N1—C1	101.4 (2)
C3—C4—C5—C6	0.1 (4)	C7—C2—N1—C1	−80.2 (3)
C4—C5—C6—C7	−0.2 (4)		

### Hydrogen-bond geometry (Å, °)

**Table:**

*D*—H···*A*	*D*—H	H···*A*	*D*···*A*	*D*—H···*A*
N1—H1···S1^i^	0.86	2.53	3.368 (2)	165
N2—H2B···S1^ii^	0.86	2.52	3.362 (2)	166

Symmetry codes: (i) −*x*+1/2, −*y*+3/2, −*z*; (ii) −*x*, −*y*+2, −*z*.
